# Developing feasible and acceptable strategies for integrating the use of patient-reported outcome measures (PROMs) in gender-affirming care: An implementation study

**DOI:** 10.1371/journal.pone.0301922

**Published:** 2024-04-16

**Authors:** Rakhshan Kamran, Liam Jackman, Anna Laws, Melissa Stepney, Conrad Harrison, Abhilash Jain, Jeremy Rodrigues

**Affiliations:** 1 Nuffield Department of Orthopaedics, Rheumatology and Musculoskeletal Sciences, University of Oxford, Oxford, United Kingdom; 2 Michael G. DeGroote School of Medicine, McMaster University, Hamilton, Ontario, Canada; 3 Temerty Faculty of Medicine, University of Toronto, Toronto, Ontario, Canada; 4 Northern Region Gender Dysphoria Service, Newcastle, United Kingdom; 5 Department of Psychiatry, University of Oxford, Oxford, United Kingdom; 6 Warwick Clinical Trials Unit, University of Warwick, Warwick, United Kingdom; 7 Department of Plastic Surgery, Stoke Mandeville Hospital, Buckinghamshire Healthcare NHS Trust, Aylesbury, United Kingdom; Florida Atlantic University Charles E Schmidt College of Medicine, UNITED STATES

## Abstract

**Objective:**

Use CFIR guidance to create comprehensive, evidence-based, feasible, and acceptable gender-affirming care PROM implementation strategies.

**Design, setting, participants:**

A 3-Phase participatory process was followed to design feasible and acceptable strategies for integrating PROMs in gender-affirming care. In Phase 1, barriers and enablers to PROM implementation for gender-affirming care were identified from a previous systematic review and our prior qualitative study. We used the CFIR-ERIC tool to match previously identified barriers and enablers with expert-endorsed implementation strategies. In Phase 2, implementation strategy outputs from CFIR-ERIC were organised according to cumulative percentage value. In Phase 3, gender-affirming care PROM implementation strategies underwent iterative refinement based on rounds of stakeholder feedback with seven patient and public partners and a gender-affirming healthcare professional.

**Results:**

The systematic review and qualitative study identified barriers and enablers to PROM implementation spanning all five CFIR domains, and 30 CFIR constructs. The top healthcare professional-relevant strategies to PROM implementation from the CFIR-ERIC output include: identifying and preparing implementation champions, collecting feedback on PROM implementation, and capturing and sharing local knowledge between clinics on implementation. Top patient-relevant strategies include: having educational material on PROMs, ensuring adaptability of PROMs, and collaborating with key local organisations who may be able to support patients.

**Conclusions:**

This study developed evidence-based, feasible, and acceptable strategies for integrating PROMs in gender-affirming care, representing evidence from a systematic review of 286 international articles, a qualitative study of 24 gender-affirming care patients and healthcare professionals, and iteration from 7 patient and public partners and a gender-affirming healthcare professional. The finalised strategies include patient- and healthcare professional-relevant strategies for implementing PROMs in gender-affirming care. Clinicians and researchers can select and tailor implementation strategies best applying to their gender-affirming care setting.

## Introduction

Gender-affirming care includes a range of psychosocial, hormonal, and surgical care offered to affirm and support a person’s experience of their gender when it is different from sex assigned at birth. Gender-affirming care is life-saving treatment, which can reduce a person’s gender dysphoria and decrease suicidality, depression, and anxiety [1]. In order to plan for and provide effective gender-affirming care that aligns with a patient’s goals, values, and priorities, the needs and experiences of the individual must be explored holistically and in a patient-centred manner [1]. Patient-reported outcome measures (PROMs) may help with this [1, 2].

PROMs are self-report questionnaires that measure how patients feel and function [3]. A few examples of diverse PROMs used across various clinical areas include: the Patient Health Questionnaire (PHQ-9) used to measure degree of depression [4]; the Oxford Hip Score to measure outcomes following total hip replacement [5]; and the World Health Organization Quality of Life instrument (WHOQOL) which measures quality of life [6]. The benefits of PROMs are well researched and include improvements in communication between patients and clinicians [7], satisfaction with care [8], health outcomes [9], the detection of issues that might otherwise go unaddressed [10], and mortality [11]. For gender-affirming care, PROMs could facilitate better patient-provider communication and shared decision-making, enable the challenging of bias and/or discriminatory practice, and assist evaluating care delivery to inform service improvement [12]. A few examples of key PROMs for gender-affirming care include the Gender Congruence and Life Satisfaction Scale (GCLS) [13] and the Utrecht Gender Dysphoria Scale (UGDS) [14]. These PROMs can be integrated in gender-affirming care as part of initial baseline assessments, to monitor patients’ progress during follow-up visits, to support shared decision-making during appointments, and to augment discussions between clinicians, and patients, in general.

Despite these benefits, PROM uptake is limited, with some clinical areas reporting that 1% of clinicians use PROMs [15–18]. Many PROM implementation initiatives fail due to a lack of evidence-based implementation strategies [19–21]. Indeed, several international bodies have called for evidence-based patient-reported outcome measure (PROM) implementation to improve gender-affirming care globally [1, 2, 22–24].

Implementation science offers established methods for categorising barriers and enablers to implementation of innovations, as well as identifying strategies for addressing the barriers and leveraging the enablers [25]. The Consolidated Framework for Implementation Research (CFIR) is an implementation science “meta-framework” that categorises barriers and enablers to implementation across five domains: outer setting, inner setting, innovation, individuals, and implementation process (Table 1) [26]. Domains are further subdivided into more specific constructs [26]. A previous systematic review [12] and qualitative study [27] conducted by our team categorized patient- and healthcare professional-reported barriers and enablers to implementing PROMs in gender-affirming care, in keeping with CFIR domains. The next step forward is to develop evidence-based implementation strategies that can address these barriers and enablers.

**Table 1:** CFIR domains and definitions from Damschroder et al. 2022 [26].

CFIR Domain	Definition
Innovation	The “thing” that is being implemented, e.g., PROMs
Inner Setting	Where the innovation is being implemented, e.g., gender clinics
Outer Setting	The context in which the Inner Setting exists, e.g., healthcare system, country
Individuals	Roles and characteristics of people involved with implementation, e.g., implementation team members, innovation deliverers (i.e., healthcare professionals), innovation recipients (i.e., patients)
Implementation Process	Sequential steps and strategies to implement the innovation

The CFIR- Expert Recommendations for Implementing Change (ERIC) tool can be used to link identified barriers and enablers to create an implementation strategy [28]. The CFIR-ERIC tool is a Microsoft Excel spreadsheet where barriers and enablers to implementation, categorised according to CFIR constructs, can be inputted. The CFIR constructs are inputted as rows, and for each of the ERIC strategies, listed as columns, the Excel spreadsheet provides an output representing the cumulative percentage of implementation experts agreeing that this ERIC strategy would be effective at addressing the barrier related to the constructs entered. The ERIC strategies were developed through a modified Delphi process, compiling 73 implementation strategies from 169 implementation experts, and has been widely applied to implementation strategy design [28, 29]. The ERIC strategies were developed first, and then later were linked to CFIR constructs via the CFIR-ERIC tool. The CFIR-ERIC tool outputs provide key evidence-based strategies which can be linked to address specific implementation barriers and enablers [30, 31]. As the outputs from CFIR-ERIC are generic in nature, it is important to tailor the strategies to a specific context for acceptability and feasibility using input from key stakeholders.

The aim of this study is to use CFIR guidance to create comprehensive and evidence-based PROM implementation strategies for gender-affirming care, which may also have potential generalizability to other clinical areas.

## Materials and methods

### Designing feasible and acceptable strategies for integrating PROMs in gender-affirming care

**Fig 1:**
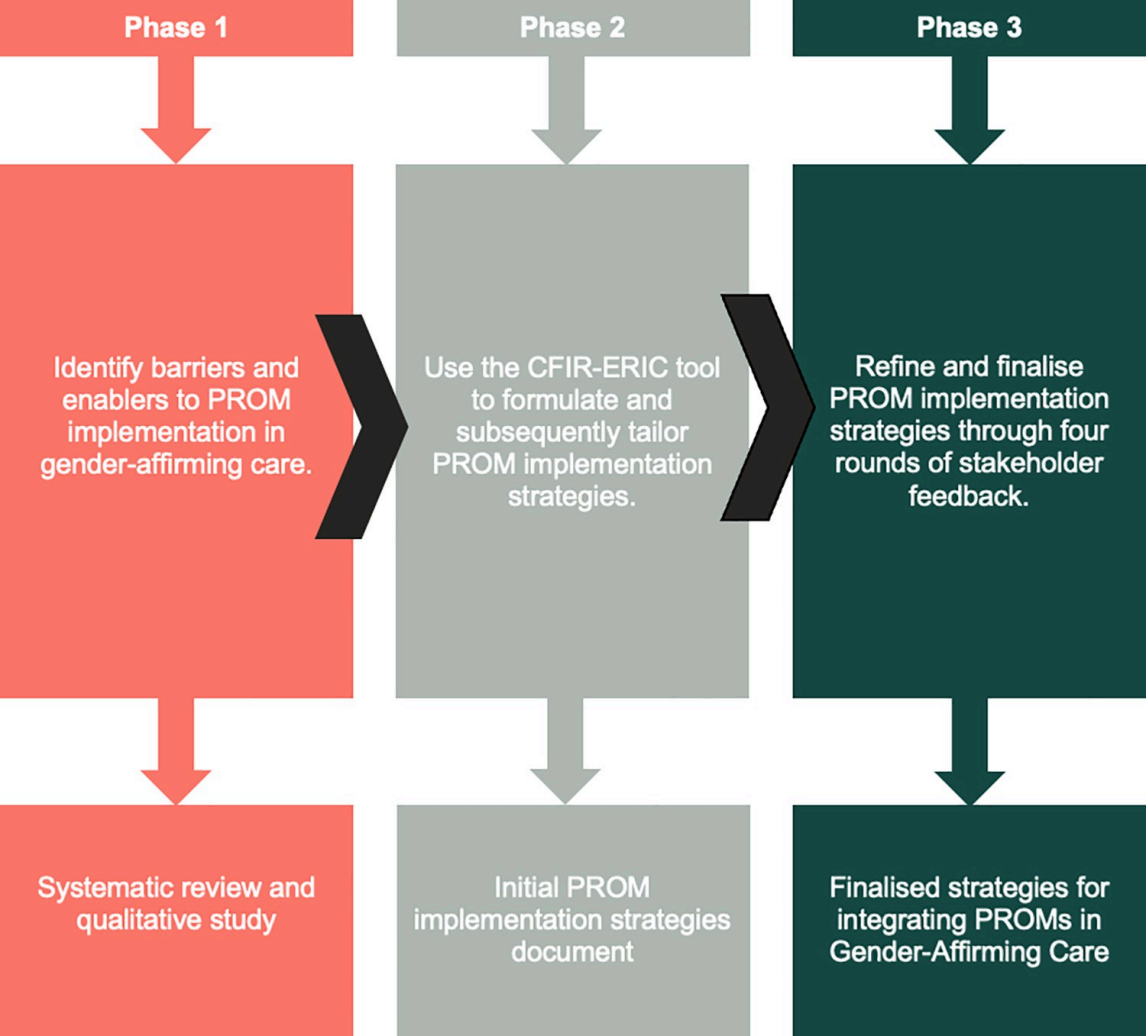
Diagram of 3-phased participatory research process to create feasible and acceptable strategies for integrating PROMs in gender-affirming care.

We followed a 3-Phase participatory process to designing feasible and acceptable strategies for integrating PROMs in Gender-Affirming Care using CFIR guidance (Fig 1).

### Phase 1

Barriers and enablers to PROM implementation for gender-affirming care were identified from our previous systematic review including 286 articles worldwide with no restrictions on date or language of publication, and our prior qualitative study that sought to understand the experiences of 14 gender-diverse patients seeking gender affirming care, and 10 interdisciplinary healthcare professionals [12, 27]. Results from the systematic review and qualitative study were organised according to CFIR construct and synthesised and prepared to be inputted into the CFIR-ERIC tool by two researchers (RK, LJ) (S1 Appendix).

### Phase 2

The data from Phase 1 were categorised with the CFIR-ERIC tool, which was used to match barriers and enablers to potential components of the implementation strategy. This was done with Excel (version 16.67) by two researchers (RK, LJ). Specifically, two researchers (RK, LJ) worked in collaboration to enter data into the CFIR-ERIC tool, which matched barriers and enablers to implementation strategies (CFIR-ERIC output is available in S2 Appendix). Implementation strategy components were organised according to cumulative percentage value from the CFIR-ERIC tool, and refined for the context of PROM implementation in gender-affirming care by two researchers (RK, LJ) (S3 Appendix). Specifically, terminology used in gender-affirming care and with PROMs was used to tailor the general statements outputted from CFIR-ERIC to the context of PROM implementation in gender-affirming care by two researchers (RK, LJ).

### Phase 3

The gender-affirming care PROM implementation strategies underwent iterative refinement based on rounds of stakeholder feedback. The gender-affirming care PROM implementation strategy developed through the CFIR-ERIC tool in Phase 2 was reviewed in sequence by seven patient and public partners representing members of the transgender and nonbinary community, and a gender-affirming care healthcare professional with expertise in PROM use for clinical practice (AL). These stakeholders were sent the gender-affirming care PROM implementation strategies and asked to provide written feedback on the acceptability and feasibility of the implementation strategies. S4 Appendix includes the feedback form used by stakeholders. The feedback from stakeholders was used to refine the implementation strategies. The implementation strategies underwent four rounds of iteration (two rounds with patient and public partners, and two rounds with a gender-affirming care healthcare professional) before all stakeholders were in consensus on the final strategies. The rounds of iteration were asynchronous: after each round of feedback, the implementation strategies were revised to respond to feedback raised. Afterwards, the revised implementation strategies were sent for another round of feedback from stakeholders. Patients and healthcare professionals were able to comment on all strategies. Consensus was reached when all key stakeholders agreed on the final implementation strategies and did not have any additional feedback to provide. Disagreements during the rounds of feedback were handled through discussion as a team. The finalised feasible and acceptable strategies for integrating PROMs in gender-affirming care represent evidence from a systematic review of 286 international articles, a qualitative study of 24 gender-affirming care patients and healthcare professionals, and input from 7 patient and public partners and a gender-affirming healthcare professional.

### Patient and public involvement

We conducted this research in partnership with seven patient and public partners, representing members of the transgender and nonbinary community. Patient and public partners were recruited through community support groups and national transgender charity organisations in the UK. Patient and public partners confirmed relevance of the research aim to create feasible and acceptable strategies for integrating the use of PROMs in gender-affirming care, and were involved in research to ensure applicability and feasibility of the implementation strategies.

### Ethics

This study was reviewed by the Clinical Trials and Research Governance Department, University of Oxford, classified as service improvement and exempt from university sponsorship or ethics committee review. This categorisation was independently ratified by the Cumbria, Northumberland, Tyne and Wear NHS Foundation Trust where the study was independently reviewed and registered: SER-22-027. Service users who had provided written consent to take part in service improvement projects with Cumbria, Northumberland, Tyne and Wear NHS Foundation Trust were contacted and invited to take part in this study. Data collection began 1 May 2023 and ended 1 August 2023.

### Reporting

Reporting follows the Standards for Reporting Implementation Studies (StaRI) guideline [32].

## Results

### Phase 1

The systematic review and qualitative study conducted previously by our team [12, 17] identified barriers and enablers to PROM implementation spanning all five CFIR domains, and 30 CFIR constructs. The systematic review and qualitative study had overlap in identifying barriers and enablers. However, the qualitative study offered additional information and explanation on the barriers and enablers to PROM implementation from the patient perspective, which was lacking in the systematic review. In summary, the key enablers identified from the systematic review and qualitative study were: adapting PROMs to be completed online and in-person, ensuring PROMs are not overly complex (too lengthy, difficult to score), ensuring PROMs are accessible to patients (i.e., those with sight issues, neurodivergence, intellectual disabilities), having a process in place to handle critical PROM responses, providing patients and healthcare providers information on what PROMs are and why they are important, and identifying implementation team members at the clinic who can facilitate implementation. S1 Appendix provides the complete list of barriers and enablers to PROM implementation for gender-affirming care identified from our previous systematic review and qualitative study.

### Phase 2

The implementation strategies outputted by the CFIR-ERIC organized by cumulative percent are available in S2 Appendix. S3 Appendix displays the implementation strategies tailored for the context of gender-affirming care. The top healthcare professional-relevant enablers include: identify and preparing implementation champions, collect feedback on PROM implementation, and capture and share local knowledge between clinics on PROM implementation. The top patient-relevant enablers include: having educational material on PROMs, ensuring adaptability of PROMs, and collaborating with key local organisations who may be able to support patients to complete PROMs.

### Phase 3

The finalised strategies for integrating PROMs in gender-affirming care are available in below (Tables 2 and 3). These tables detail patient- and healthcare professional-relevant strategies for implementing PROMs in gender-affirming care, organised into two tables (one table outlines patient-relevant strategies, and the other outlines healthcare professional-relevant strategies). Each row for both tables details a PROM implementation strategy which was created using evidence from a systematic review, qualitative study, and iterative refinement with patients and gender-affirming healthcare professionals.

**Table 2:** Patient-Relevant strategies for integrating PROMs in gender-affirming care.

Patient-Relevant Strategies
Have **educational material** (mixture of videos, animations, written information) accessible which explains: what PROMs are, why they are being implemented, how they may benefit patient care, how they work, how data will be handled, and that care access will not be jeopardised with PROM completion. Care should be taken to ensure material is not too onerous. Coproduce educational material with service users to help with accessibility and increase engagement.
Ensure the PROM selected for implementation can **adapt to patient needs** (i.e., large-print, high contrast versions, different languages).
Have **contact information provided of organisations or key individuals** who may be able to support patients to complete PROMs (i.e., Citizens Advice, Support Worker, Assistant Psychologist).
Confirm **when patients would prefer to complete PROMs** (i.e., before a clinic appointment, after a clinic appointment, in between appointments), and **where they would prefer to complete PROMs** (i.e., at home, in clinic) prior to having a PROM sent to them. Also **confirm how patients would like to receive communication** about completing PROMs (such as reminders) (i.e., through email, text message, post).
Ask patients for **feedback** on a regular basis (e.g., annually) for how PROM implementation is going and suggestions for improvement. Seek permission from patients prior to asking for feedback on PROM implementation. Where possible, gather input from patients at service evaluations in conjunction with PROM implementation feedback. Ensure patient feedback is from diverse populations.
Confirm **who patients would like PROM data to be shared with**. Allow patients to choose levels of data usage and sharing as part of the consent process (i.e., I do not consent for you to use my data for research use, but you can use it for service level feedback and for my clinician to see if I am in distress).
Have a **dedicated and private space** to complete the PROM in clinic as an option.
Have **multi-factor authentication** set up for electronic PROMs so that patients can securely and remotely access their PROM and so that it cannot be accessed by unintended recipients.
Conduct an **information session** specifically about PROM completion and data use so patients can speak/air their views with clinicians/assistants/peer support about any questions or misgivings.
Implement a **parallel system for monitoring waiting list patients** and outcomes resulting from waiting lists where possible.
Have **peer support staff available** to contact if PROM completion is difficult. Also consider whether and how patients can access a peer support worker who is similar to the patient (i.e., age, neurodivergent, ethnicity). This may mean some of the support is provided remotely or more ad- hoc and the acceptability of this should be ascertained by and led by patients. If the PROM distress falls beyond the scope of peer support services, work in collaboration with third sector organisations like LGBT switchboard or crisis mental health services.

**Table 3:** Healthcare professional-relevant strategies for integrating PROMs in gender-affirming care.

Healthcare Professional-Relevant Strategies
Identify and prepare **implementation champions** who can help to oversee and be a point of support for PROM implementation in gender clinics. This may include Identifying and involving staff members (i.e., administrative staff, assistant psychologists) who can help to oversee PROM implementation.
**Collect feedback** on PROM implementation healthcare professionals. Have feedback collection be part of an overall feedback system.
Develop and provide **educational material** to healthcare professionals on what PROMs are, why they are being implemented, how they may benefit service provision, how scoring works, and how data will be handled. Use a variety of formats such as videos, animations, written materials, and information sessions. **Co-produce educational material** with healthcare professionals to increase acceptability and engagement. Address staff responsibility for both healthcare improvement and integrity with data processing and collection. Aim to have material communicated in a ‘common language’ and part of a therapeutic strategy.
**Capture and share local knowledge** between clinics on how PROM implementation is going.
**Assess/confirm patient accessibility** needs to adapt PROMs as needed (i.e., large-print, high contrast versions, providing overlays, different languages).
**Inform higher-level leaders** (i.e., senior managers of the trust) of the PROM implementation strategy for trust-level buy in.
I**nvolve local organisations** as points of support to aid PROM implementation (e.g., Citizens Advice as a point of support to patients who may need help filling in a form, ethnically diverse local organisations). **Survey local organisations** to see if they would be willing to be involved and if they have the knowledge required to support a gender-affirming care PROM implementation effort.
I**nvolve local patient advisory groups** as points of contact to provide support on PROM implementation. This could include tailoring PROM implementation strategies to your clinic in partnership with service users.
Organize **staff meetings** aimed at identifying a PROM to implement which is not burdensome (i.e., not too lengthy or complex to score, has a computerised adaptive test option) and formalising the PROM implementation plan. Also, organise a meeting with service users to identify measures which would be acceptable to them.
Develop a **formal implementation blueprint** for the clinic on PROM implementation.
Provide **ongoing engagement** with patients to facilitate dialogue about how PROM responses are used to improve care.
Develop **academic partnerships** to help facilitate PROM implementation and interpretation when using PROMs for research.
Have **PROM responses linked to the electronic medical record** so they are accessible online. Ensure patient control over data access and where patients consent.
Develop a **process to handle critical PROM responses and feedback**. Have details of this process available for patients.

## Discussion

This study has developed feasible and acceptable strategies for integrating the use of PROMs in gender-affirming care (Tables 2 and 3) which can be used by clinicians interested in implementing PROMs for their gender-affirming care setting globally. Global clinical guidelines and international studies suggest that PROMs are essential for measuring patient outcomes of gender-affirming care [1, 12]. In the UK in particular, there is an urgent need for improving patient outcomes and relationships/trust with clinicians. We followed organised methods and specific models and processes for developing the PROM implementation strategies as described by CFIR. The strategies developed from this study can be distributed to and used by clinicians and researchers to select and tailor implementation strategies best applying to their setting. Increased clinician training to raise awareness of these strategies may also help to increase skill development to maximize use and uptake of strategies. The strategies outlined can be used as a checklist to ensure a gender-affirming care clinic is maximising potential for PROM implementation. The strategies can also be used to guide a staff meeting on implementing PROMs for a specific gender-affirming care setting.

Past research on PROM implementation has focused on other clinical areas, such as an integrated pain network [33], outpatient medical oncology [34], general outpatient clinics [35], and primary care [36]. In past research, CFIR was successfully used to plan and assess PROM implementation and linking barriers and enablers to identified implementation strategies [37]. The constructs of acceptability and feasibility were evaluated by key stakeholders when developing past PROM implementation strategies in other clinical areas [38, 39]. Recommendations have also been made in a review of PROM implementation for PROM implementation strategies to be co-developed with clinicians and patients [37]. Our study provides the first set of feasible and acceptable implementation strategies for PROM implementation for the clinical area of gender-affirming care. The PROM implementation strategies developed from our study follows recommendations from past research and uses evidence-based and implementation science theory-, model- and framework-informed methods [26, 40–42].

The implementation strategies developed from this study has implications for policy, clinical practice, and research globally. Commissioners and policy-makers can use the strategies to inform PROM implementation policy for gender-affirming care. In clinical practice, our strategies can be used to help ensure gender-affirming care aligns with patient needs that leads to urgently needed improvements in care. Some of our findings may also be of interest to researchers aiming to minimise missing data for PROMs and improve PROM response rate for studies [43].

Strengths of this study include developing a theory-, model-, and framework-informed approach to developing implementation strategies to improve PROM uptake, in line with evidence-based recommendations for implementation studies in this area [37]. Our study followed established approaches in implementation science, along with established strategy development and reporting guidelines [28, 32]. The implementation strategies from this study considered diverse and international perspectives, informed by a systematic review representing 286 studies and 85, 395 patients worldwide, and an in-depth qualitative study representing 14 patients and 10 interdisciplinary healthcare providers. Further, each phase of this research was conducted in partnership with seven patient and public partners representing the gender-affirming care community.

Limitations of this study is a lack of racial and ethnic diversity in the patient qualitative sample [27]. Future research should aim to assess the appropriateness and feasibility of the implementation strategies with ethnically diverse patient groups. Secondly, the implementation strategies developed from this study must be specified and operationalized to enable implementation for each setting. Proctor’s guidance [44] can be used to enable this for future implementation work around PROMs for different gender-affirming care settings.

## Conclusion

This study presents evidence-based, feasible, and acceptable strategies for integrating the use of PROMs in gender-affirming care. The developed strategies can be used by clinicians, policy-makers, and researchers to lead PROM implementation efforts for gender-affirming care with potential generalisability to other clinical areas. The strategies can be used to enhance patient-centeredness of gender-affirming care, as emphasised from international standard of care, and ensure PROM benefits are realised while minimising research waste associated with lack of PROM uptake.

## Supporting information

S1 Appendix Synthesised barriers and enablers to PROM implementation for gender-affirming care organised by CFIR domain. (DOCX)

S2 Appendix CFIR-ERIC output. (XLSX)

S3 Appendix Tailored gender-affirming care PROM implementation strategies. (DOCX)

S4 Appendix Feedback form for acceptability and feasibility of PROM implementation strategies. (DOCX)
