# Supplementary material for: Developing feasible and acceptable strategies for integrating the use of patient-reported outcome measures (PROMs) in gender-affirming care: An implementation study
Source: PLoS One. 2024 Apr 16;19(4):e0301922. doi: 10.1371/journal.pone.0301922 (PMC11020962; doi:10.1371/journal.pone.0301922)
Supplement: S1 Appendix — (DOCX) [file pone.0301922.s001.docx]

| **CFIR Domain** | **CFIR Construct** | **Sub-Construct** | **Description on Barrier** |
| --- | --- | --- | --- |
| **Innovation** | Adaptability | PROM | Lack of ability to have hybridized implementation (available to complete online and in-person). |
|  | Complexity | PROM | PROMs being too lengthy, complex, and confusing to interpret and score; uncertainty of when to administer PROMs; and online survey software failing. |
|  | Evidence Strength and Quality | PROM | High variability of PROMs used and lack of standardisation, using PROMs that do not demonstrate psychometric and content validity, and PROMs administered for too short a follow-up time limits incorporation of results into clinical decision making. |
|  | Innovation Cost |  | The costs of: the clinician’s time, paper and postage, online system set-up, and costs to the clinic’s reputation if something goes wrong with PROM implementation. |
|  | Innovation Design | Accessibility | Lack of PROM accessibility to people who do not have English as a first language, people with dyslexia, neurodivergence, sight issues, and intellectual disabilities. |
|  | Innovation Source | PROM | Patient mistrust with PROMs due to the sensitive nature of the questions and lack of trust that PROMs will help care provision. |
|  |  | Score | Mistrust that scoring will impact care access and patient worry that PROM scores will be shared with people they would not like to see their results. |
|  | Relative Advantage | PROM | Lack of PROM uptake for clinics offering gender-affirming care contributing to limited ability to compare outcomes and failing to fully realise PROM benefits. |
| **Outer Setting** | Critical Incidents | Responses | Uncertainty of how to handle PROM responses if a patient indicates they are not doing well and does not have a regularly scheduled appointment to follow-up on a critical PROM response. |
|  | External Pressure | Performance Measurement Pressure | Lack of interest in implementing PROMs if they will not be used to hold the health system and clinicians accountable to providing high-quality care. |
|  | Local Attitudes | Experience | Some patients reporting completing a PROM is a dehumanizing experience. |
|  |  | GP | Some patients indicating they would not complete a PROM due to worry that their responses will be shared with their GP and impact care received by their GP. |
|  | Local Conditions | Political Environment | Issues establishing trusting relationships between clinicians and patients due to the negative political environment with gender-affirming care. |
|  | Needs & Resources of Those Served by the Organization |  | Lack of implementation success when the clinic does not view measuring patient voice as a priority or helpful for care provision and treatment decision making, does not have regular quality improvement initiatives, and does not have a process which allows for PROM integration. |
| **Inner Setting** | Access to Knowledge and Information | Benefits to Service Provision | Lack of access to information demonstrating the benefits PROMs bring to service provision. |
|  | Available Resources | Time | Limited time to administer, complete, score, and interpret PROMs. |
|  | Communications | PROM Information | Lack of information with healthcare professionals and patients on what PROMs are, why PROMs are being administered, how PROM responses impact care, how PROMs do not have political alignment/a political agenda, how PROMs benefit patients; and lack of communication between physicians and patients on explaining PROMs. Another barrier was if communication on PROM information was only done through written forms (no option for a video on key information regarding PROMs). |
|  |  | Tailoring | Failing to assess how patients would like to be communicated with regarding PROMs and reminders to complete PROMs (i.e., email, text message, post). |
|  | Culture | PROM | Patients feeling uncomfortable answering sensitive questions due to past experiences of discrimination in healthcare settings and lack of clinic culture of regular PROM use. |
|  |  | Recipient-Centeredness | Lack of process to ensure PROM/PROM information is not sent to unintended recipients and accidentally outs a patient. |
|  | Implementation Climate | Compatibility | Lack of the ability of PROMs to be updated to current terminology in gender-affirming care to reflect current standards. |
|  |  | Relative Priority | Lack of clinicians believing measuring outcomes is a priority. |
|  |  | Tension for Change | Lack of organisational climate aiming to want to better understand patient experiences, improve patient monitoring, and compare between treatments. Also, not having a trans advisory board for gender clinics. |
|  | Structural Characteristics | Physical Infrastructure and Information Technology Infrastructure | Not having physical space available to complete PROMs and not having an electronic system available to administer PROMs. |
|  | Readiness for Implementation | Leadership Engagement | Lack of clinician leaders engaged and ready to implement PROMs. |
| **Individuals** | High-level Leaders | Senior Management | Failing to have senior management buy-in is a barrier to implementation as it limits PROM implementation being seen as a “legitimate” strategy that is worth buying into. |
|  | Implementation Facilitators |  | Lack of staff helping to facilitate implementation (i.e., not having support from key individuals who might be able to help like administrative staff or assistant psychologists). |
|  | Implementation Team Members | Peer Support | Clinics that do not have a peer support system in place to connect patients with if completing PROMs is distressing. |
|  | Individual Identification with the Organization | Gender Clinic | People reluctant to visit the hospital/clinic due to association of life pre-transition, people afraid of the disclosure of their former identity being revealed through PROM completion, and people wanting their transition to be private. |
|  | Knowledge & Beliefs about the Innovation | PROM | Belief that Likert-based PROMs do not capture the emotional and social complexities related to gender transition and belief that PROMs view gender as too binary. |
|  | Other Implementation Support | Organisations | Failing to connect patients with local organizations that may be able to help them complete the PROM. |
| **Implementation Process** | Assessing Needs | Innovation Recipients | Failing to assess the needs of patients, including accessibility needs to adapt the PROM to a certain format and failing to assess how often patients would like to complete PROMs. |
|  | Engaging | Key Stakeholders | Not having members of the transgender and nonbinary community included in creating a PROM implementation strategy. |
|  |  | Innovation Deliverers | Limited engagement with clinicians involved with PROM implementation and communicating with patients about PROMs. |
|  |  | Innovation Recipients | Issues with engagement with people who are non-White, older age, lower socioeconomic status, living in a rural location, and those who have not completed higher education. Difficulty engaging participants who may move to a different city and change their name post-transition. Limited engagement with patients due to feeling they will not gain from PROMs. |
|  | Executing | Data Collection | Lack of standardisation for data collection impacting interpretability of PROM results. |
|  | Reflecting & Evaluating |  | Not having PROM implementation be a continuous and iterative process informed with regular input from service users. |
|  | Tailoring Strategies | Timing | Timing PROM administration at certain times (i.e., after a distressing clinic appointment). |

Appendix 2. Synthesised barriers and enablers to PROM implementation for gender-affirming care organised by CFIR domain
