# Supplementary material for: Developing feasible and acceptable strategies for integrating the use of patient-reported outcome measures (PROMs) in gender-affirming care: An implementation study
Source: PLoS One. 2024 Apr 16;19(4):e0301922. doi: 10.1371/journal.pone.0301922 (PMC11020962; doi:10.1371/journal.pone.0301922)
Supplement: S3 Appendix — (DOCX) [file pone.0301922.s003.docx]

| **Tailored Patient-Relevant Strategies** | **Generic CFIR-ERIC Strategy Output(s)** |
| --- | --- |
| Having educational material accessible to me which explains what PROMs are, why they are being implemented, how they may benefit my care, how they work, and how data will be handled. | “Conduct educational meetings”  “Develop educational materials”  “Distribute educational materials” |
| Being able to adapt the PROM to my needs (i.e., large-print, high contrast versions, being provided overlays) | “Promote adaptability” |
| Have contact information provided to me of organisations who may be able to support me to complete PROMs. | “Identify and prepare champions” |
| Have multi-factor authentication set up so that I can securely and remotely access my PROM and so that it cannot be accessed by unintended recipients. | “Provide local technical assistance”  “Facilitate relay of clinical data to providers” |
| Have my clinic ask for my feedback on a regular basis (e.g., every 6 months) on how PROM implementation is going and if I have any suggestions on how to improve it. | “Create a learning collaborative”  “Conduct local needs assessment”  “Conduct cyclical small tests of change”  “Audit and provide feedback”  “Purposely re-examine the implementation”  “Obtain and use patient/consumer feedback” |
| Being able to indicate who I would like PROM data to be shared with. | “Involve patients/consumers”  “Prepare patients to be active participants”  “Develop resource sharing agreements” |
| Having a dedicated and private space to complete the PROM in clinic as an option. | “Promote adaptability”  “Tailor strategies”  “Facilitation”  “Change physical structure and equipment” |
| Confirming how I would like to receive communication about completing PROMs (such as reminders) (i.e., through email, text message, post). | “Facilitate relay of clinical data to providers”  “Intervene with patients to enhance uptake and adherence” |
| Having peer support staff available to contact if PROM completion is distressing. | “Use advisory boards and workgroups” |
| Confirming when I would prefer to complete PROMs (i.e., before a clinic appointment, after a clinic appointment, in between appointments) prior to having a PROM sent to me. | “Facilitation”  “Tailor strategies”  “Provide ongoing consultation”  “Obtain formal commitments”  “Develop resource sharing agreements” |
| Having the option of whether I would like to complete the PROM online or in-person at the clinic. | “Tailor strategies”  “Involve patients/consumers”  “Obtain and use patients/consumers and family feedback” |
| **Tailored Healthcare Professional-Relevant Strategies** |  |
| Identify and prepare implementation champions who can help to oversee and be a point of support for PROM implementation in gender clinics. | “Identify and prepare champions” |
| Continue to collect feedback on barriers and enablers to PROM from service users and healthcare professional’s implementation for gender-affirming care to refine the implementation plan. | “Assess for readiness and identify barriers and facilitators” |
| Develop and provide educational material to patients and healthcare professionals on what PROMs are, why they are being implemented, how they may benefit service provision, how scoring works, and how data will be handled. | “Develop educational materials”  “Distribute educational materials” |
| Capture and share local knowledge between clinics on how PROM implementation is going. | “Capture and share local knowledge” |
| Assess/confirm patient accessibility needs to adapt PROMs as needed (i.e., large-print, high contrast versions, providing overlays) | “Promote adaptability” |
| Inform higher-level leaders of the PROM implementation strategy for trust-level buy in. | “Inform local opinion leaders” |
| Involve local organisations as points of support to aid PROM implementation (e.g., Citizens Advice as a point of support to patients who may need help filling in a form). | “Capture and share local knowledge”  “Build a coalition” |
| Involve local patient advisory groups as points of contact to provide support on PROM implementation. | “Use advisory boards and workgroups” |
| Organize a staff meeting on PROM implementation to identify a PROM to implement valid yet not too lengthy or complex to score. | “Conduct educational meetings” |
| Develop a formal implementation blueprint for your clinic on PROM implementation. | “Develop a formal implementation blueprint” |
| Develop and implement tools for multi-factor authentication for remote PROM completion so that PROMs are not sent and accessed by unintended recipients. | “Provide local technical assistance”  “Facilitate relay of clinical data to providers” |
| Provide ongoing engagement with patients to facilitate dialogue about how PROM responses are used to improve care. | “Involve patients/consumers”  “Obtain and use patient feedback” |
| Assess who patients would like PROM results to be shared with to allow for patient autonomy in who can access PROM data. | “Facilitate relay of clinical data to providers”  “Obtain and use patient feedback” |
| Develop academic partnerships to help facilitate PROM implementation. | “Develop academic partnerships” |
| Create a dedicated space in your clinic where patients can complete a PROM if they would like a private space. | “Promote adaptability”  “Tailor strategies”  “Facilitation”  “Change physical structure and equipment” |
| Have PROM responses link to the electronic medical record so they are accessible online. | “Facilitate relay of clinical data to providers” |
| Develop a process to handle critical PROM responses. | “Develop and implement tools for quality monitoring” |
| Confirm how patients would like to complete PROMs and receive reminders to complete PROMs (i.e., email, text message, post). | “Involve patients/consumers”  “Prepare patients to be active participants”  “Develop resource sharing agreements” |
| Involve peer support staff in PROM implementation as a point of support to patients who may find completing PROMs distressing. | “Use advisory boards and workgroups”  “Develop and implement tools for quality monitoring”  “Obtain formal commitments” |
| Confirm when patients would like to complete PROMs (i.e., before a clinic appointment, after a clinic appointment) prior to administration. | “Facilitate relay of clinical data to providers”  “Intervene with patients to enhance uptake and adherence” |
| Identify and involve staff members (i.e., administrative staff, assistant psychologists) who can help to oversee PROM implementation. | “Identify and prepare champions”  “Organize clinician implementation team meetings”  “Revise professional roles” |
| Ensuring that PROMs can be completed online or in-person based on patient preference. | “Promote adaptability”  “Facilitation”  “Change physical structure and equipment” |
