# Supplementary material for: Developing feasible and acceptable strategies for integrating the use of patient-reported outcome measures (PROMs) in gender-affirming care: An implementation study
Source: PLoS One. 2024 Apr 16;19(4):e0301922. doi: 10.1371/journal.pone.0301922 (PMC11020962; doi:10.1371/journal.pone.0301922)
Supplement: S4 Appendix — (DOCX) [file pone.0301922.s004.docx]

The following tables outline patient and healthcare professional-relevant PROM implementation strategies for gender-affirming care. Please provide your thoughts on the acceptability and feasibility of each strategy, if any strategies should be removed or added, and your general thoughts. Thank you!

| **Patient-Relevant Strategies** | **Comments on Acceptability** | **Comments on Feasibility** | **General Comments** |
| --- | --- | --- | --- |
| 1. Having educational material accessible to me which explains what PROMs are, why they are being implemented, how they may benefit my care, how they work, and how data will be handled. |  |  |  |
| 1. Being able to adapt the PROM to my needs (i.e., large-print, high contrast versions, being provided overlays) |  |  |  |
| 1. Have contact information provided to me of organisations who may be able to support me to complete PROMs. |  |  |  |
| 1. Have multi-factor authentication set up so that I can securely and remotely access my PROM and so that it cannot be accessed by unintended recipients. |  |  |  |
| 1. Have my clinic ask for my feedback on a regular basis (e.g., every 6 months) on how PROM implementation is going and if I have any suggestions on how to improve it. |  |  |  |
| 1. Being able to indicate who I would like PROM data to be shared with. |  |  |  |
| 1. Having a dedicated and private space to complete the PROM in clinic as an option. |  |  |  |
| 1. Confirming how I would like to receive communication about completing PROMs (such as reminders) (i.e., through email, text message, post). |  |  |  |
| 1. Having peer support staff available to contact if PROM completion is distressing. |  |  |  |
| 1. Confirming when I would prefer to complete PROMs (i.e., before a clinic appointment, after a clinic appointment, in between appointments) prior to having a PROM sent to me. |  |  |  |
| 1. Having the option of whether I would like to complete the PROM online or in-person at the clinic. |  |  |  |

| **Should any strategies be added or removed?** |
| --- |
|  |

| **Any other comments?** |
| --- |
|  |

| **Healthcare Professional-Relevant Strategies** | **Comments on Acceptability** | **Comments on Feasibility** | **General Comments** |
| --- | --- | --- | --- |
| 1. Identify and prepare implementation champions who can help to oversee and be a point of support for PROM implementation in gender clinics. |  |  |  |
| 1. Continue to collect feedback on barriers and enablers to PROM from service users and healthcare professional’s implementation for gender-affirming care to refine the implementation plan. |  |  |  |
| 1. Develop and provide educational material to patients and healthcare professionals on what PROMs are, why they are being implemented, how they may benefit service provision, how scoring works, and how data will be handled. |  |  |  |
| 1. Capture and share local knowledge between clinics on how PROM implementation is going. |  |  |  |
| 1. Assess/confirm patient accessibility needs to adapt PROMs as needed (i.e., large-print, high contrast versions, providing overlays) |  |  |  |
| 1. Inform higher-level leaders of the PROM implementation strategy for trust-level buy in. |  |  |  |
| 1. Involve local organisations as points of support to aid PROM implementation (e.g., Citizens Advice as a point of support to patients who may need help filling in a form). |  |  |  |
| 1. Involve local patient advisory groups as points of contact to provide support on PROM implementation. |  |  |  |
| 1. Organize a staff meeting on PROM implementation to identify a PROM to implement valid yet not too lengthy or complex to score. |  |  |  |
| 1. Develop a formal implementation blueprint for your clinic on PROM implementation. |  |  |  |
| 1. Develop and implement tools for multi-factor authentication for remote PROM completion so that PROMs are not sent and accessed by unintended recipients. |  |  |  |
| 1. Provide ongoing engagement with patients to facilitate dialogue about how PROM responses are used to improve care. |  |  |  |
| 1. Assess who patients would like PROM results to be shared with to allow for patient autonomy in who can access PROM data. |  |  |  |
| 1. Develop academic partnerships to help facilitate PROM implementation. |  |  |  |
| 1. Create a dedicated space in your clinic where patients can complete a PROM if they would like a private space. |  |  |  |
| 1. Have PROM responses link to the electronic medical record so they are accessible online. |  |  |  |
| 1. Develop a process to handle critical PROM responses. |  |  |  |
| 1. Confirm how patients would like to complete PROMs and receive reminders to complete PROMs (i.e., email, text message, post). |  |  |  |
| 1. Involve peer support staff in PROM implementation as a point of support to patients who may find completing PROMs distressing. |  |  |  |
| 1. Confirm when patients would like to complete PROMs (i.e., before a clinic appointment, after a clinic appointment) prior to administration. |  |  |  |
| 1. Identify and involve staff members (i.e., administrative staff, assistant psychologists) who can help to oversee PROM implementation. |  |  |  |
| 1. Ensuring that PROMs can be completed online or in-person based on patient preference. |  |  |  |

| **Should any strategies be added or removed?** |
| --- |
|  |

| **Any other comments?** |
| --- |
|  |
